# Conceptualizing the health and well-being impacts of social enterprise: a UK-based study

**DOI:** 10.1093/heapro/dax009

**Published:** 2017-03-28

**Authors:** Bobby Macaulay, Michael J Roy, Cam Donaldson, Simon Teasdale, Alan Kay

**Affiliations:** 1 Yunus Centre for Social Business and Health, Glasgow Caledonian University, Cowcaddens Road, Glasgow, UK; 2 Glasgow School for Business and Society, Glasgow Caledonian University, Cowcaddens Road, Glasgow, UK

**Keywords:** UK, social enterprise, public health, conceptual modelling

## Abstract

Social enterprises–businesses that work for social benefit rather than for the maximization of financial returns to shareholders or owners–could potentially prove to be an innovative and sustainable way of tackling ‘upstream’ social determinants of health. However, empirical work focusing upon how, and to what extent, social enterprise-led activity may impact upon health and well-being is still relatively scarce. This study examines how social enterprises portray their impact, and how such impacts may be considered in health and well-being terms. Through analysing evaluative reports of the work of social enterprises in Scotland (*n* = 17) utilizing a ‘process coding’ method, we investigate both the self-reported impacts of the work of social enterprises and the mechanisms by which these are said to be derived. Revisiting previous conceptualizations in the extant literature, this work allows us to present an ‘empirically-informed’ conceptual model of the health and well-being impacts of social enterprise-led activity, and thus presents a significant advance on previous hypothetical, theoretically-based conceptualizations. It is considered that these findings further improve our overall knowledge of ways in which social enterprise and other parts of the third sector could be considered as potentially valuable ‘non-obvious’ public health actors.

## INTRODUCTION

It has long been recognized that community-based organizations can tackle aspects of social vulnerability (Galea *et al.*, 2005) which we increasingly understand to be critical to public health. One particular form of organization that has attracted considerable policy attention, particularly in recent times, is the ‘social enterprise’–a business with social objectives, where surpluses are reinvested in social purposes, rather than for distribution to shareholders or investors (Galera and Borzaga, 2009). There are a number of prominent examples of social enterprises in the UK, including *The Big Issue*, *Divine Chocolate* and *Fifteen*–the restaurant founded by celebrity chef Jamie Oliver which provides opportunities for young people at risk of social exclusion. However, most social enterprises are small in scale, owned and operated by, and for the benefit of, local communities (Ridley-Duff and Bull, 2015).

When social enterprise has been discussed in relation to public health, it is most often in relation to its role or potential as a mechanism for *delivering* health and social care services, either as an alternative, or complement, to mainstream provision (Hazenberg and Hall, 2014) or as a mechanism for enhancing community involvement and service design, particularly in rural contexts (Muñoz, 2011; Farmer *et al.*, 2012). However, there is still a significant gap in knowledge of how, and to what extent, such activity can impact upon the social determinants of health, particularly in relation to health-enhancing mechanisms and causal pathways.

Drawing upon externally audited reports of the activities and effects of the work of social enterprises, this paper aims to address this perceived gap by investigating the impacts of social enterprise-led activity and the mechanisms through which such impacts are said to be derived. Reflecting upon extant theoretical conceptualizations, our findings are then used to inform the development of a new ‘empirically informed’ conceptual model, and we conclude by reflecting on possible future research directions for researching the social enterprise/public health nexus.

## BACKGROUND

The idea that organizations led by social entrepreneurs may have a vital role to play in the development of health promotion through community-based action on the social determinants of health was mooted as far back as the end of the 1990s in the context of the Healthy Cities initiative (Catford, 1998; De Leeuw, 1999). Since then, the body of literature relating to the health and well-being impact of social enterprise-led activity has slowly developed, from grey literature written mainly by practitioners (McDermid *et al.*, 2008; Boswell *et al.*, 2009; Westwater, 2009) to theoretical or conceptual papers written by academics (Roy *et al.*, 2013, 2015b, 2017; Farmer *et al.*, 2012; Muñoz *et al.*, 2015), and we have only now reached a point where we are starting to see systematic reviews emerging. The recent systematic review by Mason *et al.* (2015) sets social enterprise within a wider context–as a form of ‘social innovation’–and attempts to assess the ability of such innovations to address health equity, finding inconsistent evaluative evidence of impact. Some of the benefits they are able to identify, however, include ‘the mobilization of latent or unrealized value through new combinations of (social, cultural and material) resources; growing bridging social capital and purposeful approaches to linking individual knowledge and experience to institutional change’ (Mason *et al.*, 2015: ii116).

Roy *et al.* 2014, meanwhile, set out their hypothetical case for social enterprises–*all* social enterprises–to potentially be considered as a complex form of public health ‘intervention’ since they work to address aspects of social vulnerability at the local level, irrespective of whether they explicitly *intend* to have a health impact. An important gap in evidence remains, however, in relation to empirical work which seeks to test this hypothesis. This paper aims to contribute towards filling that gap, to explore how social enterprise practitioners–implicitly or explicitly–conceptualize the impact of their activities upon the health and well-being of the individuals and communities they seek to support. From this analysis, we construct and present an ‘empirically informed’ conceptual model in order to provide a platform for future enquiry.

One method of gaining an insight into the various ways in which practitioners explain their impact is through an assessment of reports developed specifically for such purposes, namely ‘social impact measurement’ reports. Social impact measurement has had a chequered history as social enterprises have come under increasing pressure to evidence the ‘social value’ (Di Domenico *et al.*, 2010) that they purport to create, particularly as a means of gaining legitimacy from stakeholders and funders (Arvidson and Lyon, 2014). The two most common methods of social impact measurement in the UK, among a great many that have proliferated in recent years, are Social Accounting and Audit (SAA) and Social Return on Investment (SROI) (Gibbon and Dey, 2011). Both methods share a number of similarities in that they: both undertake processes to account for activities of the organization; seek to incorporate the voices of a broad range of stakeholders; and consider the ways in which each activity impacts upon social, environmental and economic factors (Gibbon and Dey, 2011; Hall and Arvidson, 2014). In many cases their reports are then ‘audited’ (SAA) or ‘assured’ (SROI) by an external observer to independently assess the validity of the claims being made. Thus, these reports provide a valuable insight into the intentions, activities and perceived outcomes of social enterprise practitioners, data which we could then analyse in terms of their potential health and well-being impacts.

## METHODOLOGY AND METHODS

SAA reports were sourced from the Social Audit Network website^1^ (108 reports) while SROI reports were sourced from the Members’ Area of the SROI Network website^2^ (59 reports), which has since been renamed Social Value UK. Only ‘assured’/‘audited’ reports were included in the analysis to lend a degree of quality, accuracy and external validity. Given that conceptions of social enterprise are ‘politically, culturally, historically and geographically variable’ (Teasdale, 2012, p. 100), we also decided to limit our sample to organizations based in a single polity with which we have significant knowledge, namely Scotland. Widespread acceptance of the definition of social enterprise by government and the sector means that Scotland has relatively coherent boundaries around the social enterprise concept. This definition requires organizations to aspire towards financial independence through trading, and contain an ‘asset lock’, meaning ‘profits are reinvested in the business or in the beneficiary community and not distributed to owners/shareholders/investors’ (Senscot, 2010).

Scotland has recently been described by Scottish Government politicians as having ‘the most supportive environment in the world’ for social enterprise (Roy *et al.*, 2015a) and a recent report (Social Value Lab, 2015) estimated that there are 5199 social enterprises currently operating in Scotland, employing 112 409 individuals, turning over £3.6 Billion per annum, and holding £8.77 Billion in assets.

87 SAA reports and 46 SROI reports were disregarded as the organizations were based outside Scotland, while 7 SAA and 4 SROI were excluded for lacking a significant trading element. The remaining reports were examined in greater depth to establish whether their activities and institutional form met the accepted definition of social enterprise as detailed above, with 5 SAA and 1 SROI not containing the requisite ‘asset-lock’. In the end, 17 reports (9 SAA, 8 SROI) were analysed. Data found in these reports, in addition to publically available information from the Office of the Scottish Charity Regulator (OSCR) and the Financial Conduct Authority (FCA), were used to inform details on the sample as shown in Table 1.

**Table 1:** Details of sample social enterprises

	Organization	Location	Brief description	Constitutional form	Report	Published	Period covered
1	BRAG Enterprises	Central fife	Aims to drive the social and economic regeneration of communities in Fife and around Scotland through the facilitation of employment, training and business development opportunities.	Company limited by guarantee with charitable status	SAA	2004	2003–2004
2	Cranhill Credit Union	Glasgow	Provides financial services to residents of the ‘common bond’ area in the East End of Glasgow in an effort to alleviate poverty and disadvantage.	Industrial and Provident Society	SAA	2005	2003–2005
3	Easthall Residents Association	Glasgow	Provides a community facility where residents can access services related to housing, employment and training while promoting community engagement through groups and recreational activities.	Company limited by guarantee with charitable status	SAA	2005	2004–2005
4	Milltown Day Workshops	Laurencekirk, Aberdeenshire	Facilitates opportunities for adults with mental disabilities and learning difficulties to take part in productive employment for the benefit of themselves and the local community	Company limited by guarantee with charitable status	SAA	2005	2003–2004
5	Scotwest Credit Union	Glasgow	Provides financial services to a broad range of individuals who may otherwise be excluded from mainstream banking provision.	Industrial and Provident Society	SAA	2008	2007–2008
6	The Wise Group	Scotland	Integrates individuals who experience barriers to entering the job market into productive employment with a view to helping them to enter the mainstream employment market.	Company limited by guarantee with charitable status	SAA	2008	2007
7	West Whitlawburn Housing Co-operative	South Lanarkshire	Offers affordable housing to individuals and families while also providing access to services and community facilities and advocating for greater opportunities for the development of the surrounding area.	Industrial and Provident Society with charitable status	SAA	2009	2008–2009
8	Banff and Macduff Community Trust	Aberdeenshire	Aims to develop community spirit and collective pride among the local community, while making the area more attractive to businesses and tourists.	Company limited by guarantee with charitable status	SAA	2010	2009–2010
9	Scotia Clubhouse	Glasgow	Provides opportunities for people recovering from mental health problems to engage in education and employment opportunities.	Unincorporated association	SROI	2010	2010
10	Auchinleck Community Development Initiative	East Ayrshire	Uses gardening as a means to develop community interaction, learning and training opportunities and employment integration, as well as an opportunity for local people to buy fresh produce.	Company limited by guarantee with charitable status	SROI	2011	2010–2011
11	Cunninghame Housing Association	Saltcoasts, North Ayrshire	Provides affordable housing to individuals and families while attempting to regenerate the social, economic and environmental situation in Saltcoats.	Industrial and Provident Society with charitable status	SROI	2011	2010
12	Gorgie City Farm	Edinburgh	Facilitates education and employment opportunities while providing a safe environment for families and vulnerable individuals to interact and learn about nature.	Company limited by guarantee with charitable status	SROI	2011	2009
13	The Action Group	Edinburgh	Integrates adults with learning difficulties into employment opportunities while seeking to improve their general wellbeing and eventually integrate them into the mainstream employment market.	Company limited by guarantee with charitable status	SROI	2011	2009–2010
14	The Bread Maker	Aberdeen	Provides employment opportunities to adults with mental or physical disabilities by integrating them into different roles within a café and bakery in Aberdeen City Centre.	Company limited by guarantee with charitable status	SROI	2011	2009–2010
15	West Bridge Mill	Kirkcaldy, Fife	Provides those in need of supported or short-term accommodation the opportunity to live semi-independently with support and security to eventually re-enter conventional housing.	Company limited by guarantee with charitable status	SROI	2012	2009
16	Horizon Housing Association	West Lothian	Offers basic alterations and maintenance services to elderly or other vulnerable people who may struggle with minor but crucial repairs and adaptations to their home.	Industrial and Provident Society with charitable status	SROI	2013	2011
17	North East Sensory Services	Aberdeenshire	Provides support services and training and employment opportunities for blind and deaf adults in Aberdeenshire, encouraging social interaction and mutual support among members.	Company limited by guarantee with charitable status	SAA	2014	2013–2014

In an attempt to discover what it is that social enterprises claim to do, and what impacts they consider to be caused by such activities, data concerning the distinct processes and outcomes mentioned in each organization’s report were identified through utilizing a form of coding called Process Coding (Corbin and Strauss, 2007, pp. 96–97; Saldaña, 2013, pp. 77–81). This method identifies semantic clues within qualitative data to identify the ways in which an activity (or ‘process’) can lead to a final outcome. This can take the form of identifying gerunds (working, learning, interacting, etc.) and other distinct statements of cause and effect which could be identified within the reports. Processes, in this case, were identified as any activity of the social enterprise related to a particular health and well-being outcome, irrespective of whether or not the practitioners explicitly *intended* to have an impact along such lines. The various distinct processes were grouped into broad themes (‘themeing the data’-see Saldaña, 2013) and coherent sub-themes in order to make sense of the data. Outcomes, on the other hand, were considered to be the result of one or more of the processes of the social enterprise on individuals and/or their communities. We identified a number of specific processes linked to each outcome. Some of the impacts related to the target group of beneficiaries, while some were employees within the organization itself or members of the community. We also discuss the societal effects at the level of ‘systems’. To reflect the different recipients of these effects, impacts have been grouped in accordance with their perceived ‘level of outcome’, albeit certain outcomes were seen to be operating at different levels simultaneously.

Table 1 outlines the name, location, constitutional form and a brief description of each of the organizations, as well as the type of report analysed, what period of time it covered and when it was published. The sample includes a broad spectrum of social enterprises, covering 10 years and nine local authority areas across Scotland, and are split almost evenly between SAAs (*n* = 9) and SROIs (*n* = 8).

## FINDINGS

This section will briefly set out the context in which the various social enterprises operate and discuss the various social missions of the sample social enterprises and the range of vulnerabilities they seek to address. Following this, we consider the pathways through which the various social enterprises were seen to impact upon people’s lives through a public health lens, first considering ‘intermediate outcomes’ and then longer-term health outcomes.

### Context

One of the most common challenges cited was that of unemployment (1, 2, 6, 7, 9, 10, 11, 14) with recognition that that areas that experience high levels of unemployment do not only suffer economic consequences (7, 11, 12), but are considerably more likely to experience other negative impacts including poor physical and mental health (7, 9, 10, 15). Financial exclusion (2, 5), income deprivation (7, 10) and fuel poverty (6) were contributing factors to the social disadvantage (2) associated with a lack of financial means (6, 10). This had a knock-on effect on communities as areas developed social problems (7, 9, 11) and the demand to live there decreased (11), often due to community disrepair resulting from inadequate physical amenities such as housing (7, 11, 15, 16), community space or surrounding environment (3, 8), and a lack of community spirit or responsibility for the upkeep of the community (7). Other elements associated with poverty in a community were the levels of drug and alcohol abuse (6, 7, 9, 15) and high crime rates (3). There was also a lack of provision of services to support various ‘target groups’, including homeless (6, 7, 15), disabled and other vulnerable individuals (6, 15, 16), lone parents (6), ex-offenders (6) and asylum seekers (6). Social isolation, especially in the growing elderly population, meant that people struggled to get out and about and build a social network (2, 7, 9, 14, 15, 16, 17), negatively impacting upon confidence (17), independence (16) and, ultimately, health (15, 16, 17). Finally, there were often limited skills and capabilities held within communities, both concerning formal academic (6, 7), and other soft skills (17).

In order to mitigate these conditions, some organizations directly targeted these vulnerabilities, while others sought a more indirect route, whereby their work would impact upon other elements in the community.

Many social enterprises sought to support people to enter into (1, 3, 6, 8, 9, 10, 13, 14), or to remain in (1, 2, 4, 6, 7, 8, 11, 14), employment, volunteering (3, 8, 11), formal education (1, 3, 9, 10) or vocational training (4, 8, 10). This aim also related to maximizing economic returns both individuals (2, 3, 6, 10) and communities (1, 7, 8, 10, 11), developing soft skills (4, 10) and harnessing those strengths so that individuals and communities could play a role in their own development (8). Some social enterprises sought to create a pleasant social environment (1, 2, 3, 7) including a community support network (2) which would protect against social isolation (9), enhance community spirit (7, 8, 10) and address social stigma (9). It was also hoped that such a network could play a role in the management and ownership of the social enterprise (3, 7, 10, 15), delivering services (1, 3, 7, 11) and influencing policy and policy makers (1, 6, 17) through amplifying the voice of the community (8). Intended improvements to the physical environment included regenerating buildings and pieces of land (6, 7, 8, 12) and providing care (3, 15), recreation (3, 8), housing (3, 7, 9, 11, 15, 16) and financial (2, 5) services efficiently and effectively (5, 6, 7, 11, 14). Ambitions in terms of the continued funding (6, 8, 11, 14) and expansion of the social enterprise included both the broadening of these services (1, 2, 3, 5, 11, 14) and the incorporation of new ones (2, 3, 10) in a transparent (2), accountable (1) and ethical (2, 5, 7) manner. A broader desire was also to change environmental behaviour within the community so as to minimize environmental impact (3, 4, 5, 6). The desire to improve health incorporated mental (9), physical (16) and quality of life (7, 9) considerations through the improvement of health behaviours (10) and a focus on elements of emotional wellbeing (8), including dignity (2), self-respect (4), feeling valued and confidence (10).

### Outcomes

As detailed in the previous section, the perceived effects of the work of social enterprises were grouped into themes. These themes include: Enhanced social connectedness; Employment, employability and meaningful work; Economic impact; Enhanced confidence and self-esteem; Improved sense of meaning and control; Positive spaces and environments; Access to services; and Improved health and wellbeing. Each theme is backed up by data, such as that explained in Table 2, and we discuss each theme in turn.

**Table 2:** Data contributing to outcome groupings

Enhanced social connectedness	‘Wider Social Networks’ was a core outcome that spoke very clearly and loudly through the in depth qualitative interviews. It is the experience of working closely together with others in the Clubhouse environment that produces, time and again, increased number of friendships and social activity.9- Scotia Clubhouse	Community gardens…provide opportunities for socializing with and learning from fellow gardeners and residents that may normally be unavailable. This aids community cohesion by dissolving prejudice about race, and economic or educational status.12- Gorgie City Farm	“I have met people like me and feel less lonely. Now I have a social life. I see how others like me cope.”17- North East Sensory Services
Employment, employability and meaningful work	Real Jobs is supporting disabled people into sustained work which is important for the aims of reaching people furthest from the labour market and tackling inequality in employment13- The Action Group	The Wise Group’s target for 2007 was to support 3013 people into jobs and this target was exceeded by 20%, with 3653 people progressing into work… This was a substantial increase in job outcomes from 2006, when the organization assisted 2919 people to find employment and is part of an incremental growth in job outcomes over a 5 year period of 59%.6- The Wise Group	The scheme provides apprentices with valuable work experience and social skills required in order to access paid employment either within the bread maker, should a vacancy arise, or with an external employer.14- The Bread Maker
Economic impact	The majority of staff live in the local area and as a result of the employment that they are in, have salaries to spend with local business.8- Banff and Macduff Community Trust	The fact that their child is earning an income [through a traineeship] will influence the family income in a positive way.11- Cunninghame Housing Association	Clients also reported a level of financial benefit with the project having helped them move a more stable position: ‘Comfortable financially leading to a better state of mind.’6- Wise Group
Enhanced confidence and self-esteem	Seems more confident and initiates conversation now if she meets people from Milltown when she is out and about4- Milltown Day Workshops	Giving opportunities to long-term unemployed, boosting their self-esteem and confidence.8- Banff and Macduff Community Trust	Taking part in the training programme will make individuals feel more confident and have a sense of purpose and worth.10- Auchinleck Community Development Inititative
Improved sense of meaning and control	Through membership, acceptance and shared ownership of tasks, individuals with severe & long term mental health issues, find meaning, stability, new roles and purposeful work.9- Scotia Clubhouse	The approach aims to give people freedom to develop and live their lives as they wish, whilst learning that with this freedom comes accountability, repercussions and responsibility.15- West Bridge Mill	‘I was encouraged to learn new techniques and use aids such as a symbol cane. I have been able to travel independently and although I still get anxious at crossing roads, I have adjusted because of the support that was given to me.’17- North East Sensory Services
Positive spaces and environments	Easthall does not have a focal point that its residents could identify with, take advantage of and take pride in. The Glenburn Centre is now complete and provides a place for people to formally and informally meet and generate a positive impact on the area.3- Easthall Residents Association	Participants are able to enhance the amenity of the area in which they live and feel a sense of pride10- Auchinleck Community Development Inititative	The Community Gardens at Gorgie City Farm provide a green oasis for wildlife in an urban area. There is a pond, always full of frogs, a wildflower meadow, providing nectar and pollen for bees, lots of undisturbed corners for creatures to hibernate and many bird and bat boxes.12-Gorgie City Farm
Access to services	All were very happy with the level of service provided and the range of services available online5- Scotwest Credit Union	Since The Green Tree opened its doors, Banff town centre has more to offer local residents and visitors8- BMCT	We live in well designed sustainable places where we are able to access the amenities and services we need10- Auchinleck Community Development Inititative
Improved health and wellbeing	As a result of the skilled and fast response of concierge staff there has been 11 potentially life threatening incidents intervened in with successful outcomes during the period7- West Whitlawburn	The support on offer enables individuals to have their mental health monitored, which together with the collaborative working of both services, provides a good chance of a positive outcome.15- West Bridge Mill	Most volunteers experienced an improvement in their mental health as a result of working at the Community Garden Project.12- Gorgie City Farm

#### Enhanced social connectedness

*‘Wider Social Networks’ was a core outcome that spoke very clearly and loudly through the in depth qualitative interviews. It is the experience of working closely together with others in the Clubhouse environment that produces, time and again, increased number of friendships and social activity.’*

9- Scotia Clubhouse

The work of social enterprises was claimed to have resulted in the strengthening (10, 12, 16) and broadening (9, 12) of social networks in communities, increasing the number (6, 9, 10, 12, 16) and quality of individual relationships (6, 9, 10, 11, 12, 13, 15, 16), and bringing diverse members of communities closer together (10, 12). This was also seen to reduce pressure on other forms of formal or informal support for people (12, 14, 15, 16). The inclusion of individuals in the social groupings, activities and training related to formal employment was the main way through which this was achieved (6, 9, 12, 14). In turn, this led to the development of other life skills including teamwork (9, 10, 14), communication (10, 13, 14, 15) and coping skills (9, 13, 17), as well as the feeling of being involved in, and contributing to, the community (2, 10, 12, 14, 17) which were generally seen to derive from employment environments. Community spaces and activities provided a vehicle through which individuals could engage and contribute to their community (8, 10, 11, 12) while it was seen that improvements to housing could improve the quality of relationships within the family, and with others (11, 15, 16).

#### Employment, employability and meaningful work

*‘Real Jobs is supporting disabled people into sustained work which is important for the aims of reaching people furthest from the labour market and tackling inequality in employment’*

13- The Action Group

Much of the success in terms of getting individuals into employment was achieved through targeted employability support and training (1, 2, 3, 6, 7, 8, 9, 10, 11, 12, 13, 14, 15), including the development of soft skills (2, 9, 10) and the opportunity to volunteer (9, 10, 12). The creation of employment opportunities was predominantly achieved by developing sustainable trading entities which then employed local people (1, 3, 7, 8, 11, 17) and continued to develop their skills through workplace learning (2, 3, 4, 9, 10, 11, 12, 14). Other provisions to support individuals to re-enter employment included adequate housing (7, 11, 15), a supportive working environment (4, 9), the provision of childcare (7) and other services (1, 3, 4), and a local network of organizations who share skills and best-practice (4, 12). The development of work-related skills was also seen to have led to an increase in pride (7), dedication (12) and the feeling of being respected (10) and valued (4, 10, 12, 14).

#### Economic impact

*‘The majority of staff live in the local area and as a result of the employment that they are in, have salaries to spend with local business.’*

8- Banff and Macduff Community Trust

The increase in individual income was attributed largely to gaining employment (3, 6, 7, 8, 11, 13, 17). However, the majority of financial impacts related to increased savings, supported by the provision of financial services (2, 5, 6, 7), as well as reducing bills and debt (2, 5, 6, 8, 13) and other overheads related to inadequate housing (7, 11, 16). The ability to manage money more effectively was seen to have improved (7) and it was also perceived that having pay automatically deducted and invested in an employer-based savings plan reduced employee absenteeism (5).

The economic impact on communities centred around building a vibrant local economy (1, 7): generating (8, 10, 17), retaining (7, 8, 13) and spending (1, 2, 8, 11) money locally. This was achieved through the development of local trading entities (7, 8), improving the superficial look of the community (8) and building the skills and capabilities of local people through specialized business development services (1).

The work of social enterprises was seen to save on public expenditure. This was most widely claimed in the health sector (9, 10, 12, 13, 14, 15, 16) and unemployment services (6, 7, 8, 13). The most common cause for this was the provision of employment opportunities to individuals (6, 8, 13, 14). The provision of adequate housing (11, 15, 16) and outdoor spaces (12) was strongly correlated with savings to the health service (11, 12, 16), but was also seen to reduce the demand for the police (11, 13) and social service (13) involvement, as well as reducing council expenditure on housing services (11, 15, 16).

#### Enhanced confidence and self-esteem

*‘Seems more confident and initiates conversation now if she meets people from Milltown when she is out and about’*

4- Milltown Day Workshops

Feelings of confidence and self -esteem (1, 2, 4, 6, 8, 9, 10, 11, 13, 14), as well as related feelings of self-value (3, 8, 14), self-worth (6, 9, 10) are predominantly seen to derive from employment or inclusion in working activities. Increased social interaction and social status also led predominantly to greater confidence and self-esteem within individuals (9, 11, 17), as well as a sense of achievement from having a positive impact on others (10).

#### Improved sense of meaning and control

*‘Through membership, acceptance and shared ownership of tasks, individuals with severe & long term mental health issues, find meaning, stability, new roles and purposeful work.’*

9- Scotia Clubhouse

This effect consisted predominantly of individual capabilities and feelings including: independence (7, 10, 11, 14, 15, 16, 17), responsibility (4, 6, 12, 13, 15), meaning (9, 11), stability (9, 13) belonging (2, 9), fulfilment (14), happiness (6), motivation (9, 10, 12, 13), pride (2, 12, 14), reduced depression and stress (5, 6, 13), satisfaction (4, 9, 12), and a sense of identity (6, 9), purpose (9, 10, 11, 14) and achievement (1, 10). These were seen to derive from a variety of causes including employment (1, 2, 4, 5, 6, 9, 10, 11, 12, 13, 14), education (11, 14, 17) and social interaction (4, 9, 14). A number of housing services were also seen to help support people in feeling a sense of control, ownership and security (7, 11, 15, 16). In turn, the ability of individuals to maintain housing tenancy was seen to have been developed through a multitude of means, including improved life skills (15); the provision of professional counselling and advice (15); an improvement in financial means (2, 6, 15); the routine involved in a working environment (12); and the development of green space within the community (12). The provision of local services (1, 2, 5, 7, 10, 11, 15, 16, 17) are seen to generate similar effects with improvements in green space (10, 11, 12) and housing quality (7, 11, 16) specifically seen to reduce anxiety and increase satisfaction and self-worth.

#### Positive spaces and environments

*‘Easthall does not have a focal point that its residents could identify with, take advantage of and take pride in. The Glenburn Centre is now complete and provides a place for people to formally and informally meet and generate a positive impact on the area.’*

3- Easthall Residents Association

The perception of what made a space ‘positive’ was made up of the often overlapping factors of: improved superficial appearance (3, 8, 10, 11, 12); a place where different individuals and community groups could meet (3, 8, 10); and the improved ecological environment in the area (3, 7, 8, 10, 12). These spaces were also seen to provide safe places with reduced crime (3, 12), caused by a number of community-based initiatives including employment (8, 13), housing (11), social contact (10) and community facilities (3, 12). In turn, the ability to create a welcoming environment free from crime led to a sense of ownership and the ability to support social networks (8), secure resources (8, 10) and amplify the voice of the community (8).

With regard to the social environment, increased feelings of community pride can be attributed largely to changes to the physical appearance of a community (7, 10, 11, 12). Bringing different groups together (3, 9, 12, 17) and providing services (5, 10, 17) to all helped to break down stigma and prejudice (9, 11, 17) and build a sense of community cohesion (5, 7, 10, 12) and civic pride (8, 10, 11).

The processes generally perceived to lead to such environments were relatively few, consisting mainly of creating green spaces (3, 10, 11, 12), developing local businesses and community initiatives (3, 8, 10, 11, 12) and maintaining environmentally friendly institutional policies (4, 7, 8, 12). The attitude of the community and social enterprises towards environmental practices was seen to have changed due to taking steps to reduce waste and emissions and investing in gardening activities (4, 5, 6, 7, 10, 11, 12, 17).

#### Improved access to services

*‘All were very happy with the level of service provided and the range of services available online’*

5- Scotwest Credit Union

Awareness of, and access to, services was generally facilitated through the social enterprise providing new services such as community-based educational initiatives (1, 2, 6, 7, 11, 13, 14, 17), financial services (2, 5), employment support (6), recycling facilities (3) and the sale of fresh produce (10, 12). This sometimes took the form of a local social enterprise collaborating with other organizations (4, 8, 10) to make services more accessible to people (10, 15, 17). The availability of the service was then communicated to local people through official communications (4, 8, 10) or engagement with the local community (3, 4, 8, 10, 11). Investing in and providing sufficient housing for all, particularly of good quality (7, 11) was seen as being responsible for a decrease in homelessness (7, 11, 15).

#### Improved health and wellbeing

As well as the intermediate effects detailed above, reports indicated that the work of social enterprises was having a direct impact on the health of individuals and communities.

*‘As a result of the skilled and fast response of concierge staff there has been 11 potentially life threatening incidents intervened in with successful outcomes during the period’*

7- West Whitlawburn Housing Co-operative

The provision of a pleasant (11), safe, relaxing (12) environment, especially one outdoors (3, 10, 12), was perceived to benefit both physical (3, 14) and mental health (6, 9, 11, 12), as was being involved in a working environment (6, 9, 10, 14). Employment, and especially outdoor work, was seen to encourage healthy eating (4, 9, 10, 12), physical activity (3, 9, 10, 12), personal hygiene (16) and a reduction in the use of medication (6, 9, 12), drugs (6, 13) and alcohol (13). Increased social interaction (9, 10) and support (9, 17) was seen to have an effect on mental health, while good quality housing (7, 11) and health education (9, 10) were seen to benefit physical health. Quality of life and general wellbeing was seen to be enhanced through the provision of financial (2) and housing services (15, 16), job satisfaction (8), the improved appearance of the community (11) and spending time outdoors (10).

## DISCUSSION AND CONCLUSIONS

**Fig. 1:**
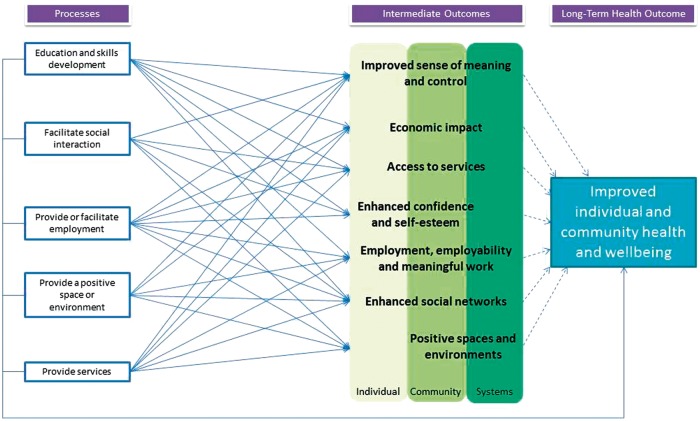
Empirically-informed conceptual model of the ways in which social enterprise-led activity impacts upon health and wellbeing.

The intention of this study was to build upon existing conceptualizations and present a new ‘empirically informed’ model upon which to base future work. Drawing on the outcomes identified in the examined reports, as well as the claimed pathways through which they were developed, the model prepared is presented at Figure 1.

In previous conceptualizations (particularly Roy *et al.*, 2014) the different types of intervention were conceived of consisting of (a) those social enterprises that involve the target beneficiaries within the work of the social enterprise, and thus could be said to *directly* intervene; and (b) those that trade in order to have an *indirect* impact upon a target set of beneficiaries. We do not explicitly distinguish between these indirect and direct impacts as we consider that a single social enterprise can have multiple impacts upon different individuals, both directly and indirectly, and at the same time. We have also attempted to convey the complexity of what is happening at the local level, recognizing that many different processes impact upon people at different levels, including at the level of the individual, the community, and broader societal systems.

There are obvious limitations of using SAAs and SROIs to construct a conceptual model. For example, there may be justifiable concerns regarding the quality of the evidence in the reports, and also who is doing the reporting, given that: (a) the outcomes were self-reported, albeit that the ones we chose were externally assessed, which injects a degree of validity; and (b) we know that many organizations use the evaluation process for promotional purposes (Arvidson and Lyon, 2014) and so may be tempted to over-claim their impact. Therefore, although not rigorously testing the extent of each impact (they are not intended to be the ‘truth’ by any means), using these claims to conceptualize and model the potential effects of social enterprises builds directly upon previous conceptual research, and can serve as a platform upon which to build future research into the health benefits of involvement with social enterprises. Potential next steps in this field of research will be to compliment these conceptual models with empirical evidence, and to develop the practical uses of these findings with regard to the evaluation of outcomes. If the activities of social enterprises can be categorized in the manner (if not precisely the groupings) listed above, the relative impacts on each of the intermediate effects, and upon health and wellbeing, may be considered in terms of a complex health intervention. Process evaluation (Moore *et al.*, 2015) may be employed to examine the relative effect of each of the potential pathways in an effort to discover which are more or less effective in regard to health improvement. This may also shed light on how social enterprise activities differ from other private, public or third sector interventions and determine whether there is anything unique about the form within the health field.

As is typical of research on the social determinants of health (particularly viz. Dahlgren and Whitehead, 1991), including in relation to contemporary discussions regarding health ‘assets’ (Morgan and Ziglio, 2007; Morgan *et al.*, 2010), we employ the terms ‘upstream’ (or distal) and ‘downstream’ (or proximal) to explain the potential impact of the identified ‘intermediate outcomes’ in health and well-being terms. It could reasonably be argued that we do not, at least not explicitly within our model, take sufficient account of socio-political factors that we know are crucial to health (Navarro, 2008; Beckfield and Krieger, 2009; Mooney, 2012; Schrecker and Bambra, 2015). Indeed, Haugh (2012) argues that social enterprise practitioners perceive the importance or value of their work in the way that they organize their activities to solve particular societal problems. Inevitably such problems are socio-political in nature: social enterprise is ‘inherently political’ (Curtis, 2015) and can even involve positing ‘an alternative economic culture that differs sharply from the market philosophy, centred instead around the provision of socially useful services, meeting need, ethical trade, and social/community empowerment and democratization’ (Amin *et al.*, 2003, p. 116). The impact of social enterprise at the level of the political economy (as far ‘upstream’ as one gets, it could be argued) also deserves further examination in the future.

Through analysing the work of social enterprises through a public health lens, we see that irrespective of whether a social enterprise considers their impacts to be explicitly ‘health-focused’, their work has clear implications for health and well-being. This study therefore advances our understanding of the role of actors that are not formally part of health systems and yet obviously have a role to play in creating the conditions for a healthy society. The intention of future research will be to explore the idea of ‘non-obvious’ public health actors in greater breadth and depth. If there is potential for social enterprises to benefit public health in developed economies, working in partnership to augment or enhance the work of well-resourced, efficient public health services, then this could even start to make an economic case for subsidy of social enterprises and for their recognition as potentially valuable public health actors. Such an eventuality may, of course, create future dilemmas. What may happen to social enterprises if they formally become part of health systems? What unintended consequences might this have for social enterprises, and for the independence of the third sector? However, if it can eventually be shown that investing in the work of actors located in the third sector, rather than in more conventional public or private health services, will yield ‘better’ results (howsoever determined) in the long term, then this may prove to have significant consequences for public health policy and practice.

## FUNDING

This work was supported by the Medical Research Council and the Economic and Social Research Council [grant number MR/L003287/1].

## Footnotes

1
www.socialauditnetwork.org.uk

2
www.socialvalueuk.org
